# An extensive molecular cytogenetic characterization in high-risk chronic lymphocytic leukemia identifies karyotype aberrations and *TP53* disruption as predictors of outcome and chemorefractoriness

**DOI:** 10.18632/oncotarget.15883

**Published:** 2017-03-03

**Authors:** Gian Matteo Rigolin, Luca Formigaro, Maurizio Cavallari, Francesca Maria Quaglia, Enrico Lista, Antonio Urso, Emanuele Guardalben, Sara Martinelli, Elena Saccenti, Cristian Bassi, Laura Lupini, Maria Antonella Bardi, Eleonora Volta, Elisa Tammiso, Aurora Melandri, Massimo Negrini, Francesco Cavazzini, Antonio Cuneo

**Affiliations:** ^1^ Hematology Section, Department of Medical Sciences, Azienda Ospedaliero-Universitaria, Arcispedale S. Anna, University of Ferrara, Italy; ^2^ Department of Morphology, Surgery and Experimental Medicine, and “Laboratorio per le Tecnologie delle Terapie Avanzate” (LTTA), University of Ferrara, Italy

**Keywords:** chronic lymphocytic leukemia, gene somatic mutations, next generation sequencing, karyotype: prognosis

## Abstract

We investigated whether karyotype analysis and mutational screening by next generation sequencing could predict outcome in 101 newly diagnosed chronic lymphocytic leukemia patients with high-risk features, as defined by the presence of unmutated *IGHV* gene and/or 11q22/17p13 deletion by FISH and/or *TP53* mutations. Cytogenetic analysis showed favorable findings (normal karyotype and isolated 13q14 deletion) in 30 patients, unfavorable (complex karyotype and/or 17p13/11q22 deletion) in 34 cases and intermediate (all other abnormalities) in 36 cases. A complex karyotype was present in 21 patients. Mutations were detected in 56 cases and were associated with unmutated *IGHV* status (*p* = 0.040) and complex karyotype (*p* = 0.047). *TP53* disruption (i.e. *TP53* mutations and/or 17p13 deletion by FISH) correlated with the presence of ≥ 2 mutations (*p* = 0.001) and a complex karyotype (*p* = 0.012). By multivariate analysis, an advanced Binet stage (*p* < 0.001) and an unfavorable karyotype (*p* = 0.001) predicted a shorter time to first treatment. *TP53* disruption (*p* = 0.019) and the unfavorable karyotype (*p* = 0.028) predicted a worse overall survival. A shorter time to chemorefractoriness was associated with *TP53* disruption (*p* = 0.001) and unfavorable karyotype (*p* = 0.025). Patients with both unfavorable karyotype and *TP53* disruption presented a dismal outcome (median overall survival and time to chemorefractoriness of 28.7 and 15.0 months, respectively). In conclusion, karyotype analysis refines risk stratification in high-risk CLL patients and could identify a subset of patients with highly unfavorable outcome requiring alternative treatments.

## INTRODUCTION

Chronic lymphocytic leukemia (CLL) is a heterogeneous disease, running an indolent course in some patients and a clinically aggressive course in others [1, 2]. Therefore, in clinical practice, prediction of outcome and response to treatment is important in an era in which several chemoimmunotherapy combinations and effective mechanism-driven treatments are available [3–5].

Prognostic/predictive factors include advanced stage [6], positivity for CD38, ZAP70 and CD49d [7–9], the unmutated configuration of the variable region of the immunoglobulin heavy chain (*IGHV*) gene [7] and specific cytogenetic lesions revealed by fluorescent *in situ* hybridization (FISH) [10]. Recent studies also demonstrated the independent negative prognostic impact of mutations of several genes, including *TP53*, *NOTCH*1 and *SF3B1* [11–15].

Next generation sequencing (NGS) techniques documented that previously unidentified genes may be mutated in CLL possibly promoting disease progression and drug resistance [16–19]. By NGS it was also demonstrated that *TP53* mutated subclones (< 15–20% of the cells) may confer the same negative prognosis as major clones (> 15–20% of the cells) detected by conventional sequencing techniques (i.e. Sanger sequencing) [20–22].

Comprehensive prognostic indexes including clinical and biological parameters were published [23, 24] and a recent systematic review [25] recommended *IGHV* and FISH analyses as useful tests for all newly diagnosed CLL patients as they may identify those 40–50% of cases with unfavorable prognosis based on the presence of an unmutated *IGHV* gene and/or 11q22/17p13 deletion [25].

Recently, karyotype aberrations were shown to represent a strong prognostic factor [26–28]. Moreover, the complex karyotype emerged as an independent predictor of inferior time to first treatment (TTFT) and shorter overall survival (OS) in patients investigated at diagnosis [29] or at disease progression [30] and in relapsed/refractory CLL treated with ibrutinib [31].

We therefore investigated whether an extended genetic characterization, including karyotype analysis using novel mitogens and mutational screening by NGS, could predict outcome in high-risk CLL patients. By correlating genetic data with clinical and biological parameters we showed that karyotype represents an independent prognostic factor for TTFT, OS and time to chemorefractoriness (TTCR).

## RESULTS

### Patients, mutational analysis and cytogenetic data

The clinical and biological characteristics of 101 high-risk CLL patients are presented in Table 1. Cytogenetic analysis showed clonal karyotype aberrations in 88/101 (87.1%) of the cases (Supplementary Table 1), a favorable karyotype in 30 (29.7%) patients, an intermediate karyotype in 36 (35.6%) and an unfavorable karyotype in 34 (33.7%) cases. A complex karyotype was present in 21 (20.8%) patients. Recurring aberrations included: 14q deletions in 12 cases, 6q deletions in 4 and 7q deletions in 3. A balanced translocation was observed in 12 patients, while 6 cases presented unbalanced translocations.

**Table 1:** Clinical and biological characteristics of the 101 CLL patients

Variable	
Age, median yrs (range)	65.6 (38.4–89.9)
Sex m/f	63/38
Binet Stage a/b/c	76/17/8
CD38 neg/pos	41/60
ZAP70 neg/pos	61/27
Normal FISH yes/no	32/69
13q14 deletion yes/no	52/49 (20/81 hierarchical)
Trisomy 12 yes/no	23/78 (21/80 hierarchical)
11q22 deletion yes/no	20/81
17p13 deletion yes/no	8/93
FISH fav/int/unfav	52/21/28
karyotype fav/int/unfav	30/36/34
Complex karyotype yes/no	21/79
*IGHV* mut/unmut	9/92
Mutated patients by NGS no/yes	45/56
N. of mutations by NGS 0/1/2/3/4	45/30/16/7/3
*TP53* disruption* yes/no	19/82
Chemotherapy yes/no	63/38
Chemorefractory yes/no	26/37
Lines of therapy 1/> 1	37/26

*17p13 deletion and/or *TP53* mutation.

Abbreviations: f, female; fav, favorable; int, intermediate; m, male; mut, mutated; neg, negative; pos, positive; unfav, unfavorable; unmut, unmutated; yrs, years.

95 somatic mutations were found in 56/101 (55.4%) cases; 80 missense mutations, 5 nonsense mutations and 10 frameshit deletions. Mutations were detected with a frequency ranging from 5.0 to 96.7% of the reads. 16 cases (15.8%) showed mutations in the *TP53* gene, 11 (10.9%) in the *NOTCH1* gene, 11 (10.9%) in the *SF3B1* gene, 8 (7.9%) in the *ATM* gene, 5 (4.9%) in the *BIRC3* gene, 5 (4.9%) in the *PTEN* gene, 4 (4.0%) in the *MYD88* gene, 4 (4.0%) in the *BRAF* gene, 4 (4.0%) in the *POT1* gene, and 18 (17.8%) cases in the remaining 11 genes (Supplementary Tables 2 and 3). 26/56 (46.4%) mutated patients presented two or more mutations.

**Table 2:** Correlations between clinical biological parameters and molecular and molecular cytogenetic results

	NGS mutations			11q22 deletion by FISH			*TP53* disruption*		
variable	yes	no	*P*	No	yes	*P*	No	yes	*P*
Age < 65/ > = 65 years	30/26	26/19	0.692	43/38	13/7	0.453	46/36	10/9	0.803
Sex m/f	33/23	30/15	0.536	51/30	12/8	0.802	51/31	12/7	1.000
Binet stage a/b-c	43/13	33/12	0.817	62/19	14/6	0.569	62/20	14/5	1.000
CD38 neg/pos	23/33	18/27	1.000	33/48	8/12	0.580	31/51	10/9	0.302
FISH fav-int/unfav	39/17	34/11	0.655	73/8	0/20	< 0.001	62/20	11/8	0.156
*IGHV* mut/unmut	8/48	1/44	0.040	8/73	1/19	0.684	1/81	8/11	< 0.001
*TP53* disruption no/yes	na	na	-	62/19	20/0	0.012	na	na	na
Muts by NGS no/yes	na	na	-	36/45	9/11	1.000	43/39	9/10^	0.800
n. of muts by NGS no/1/≥2	na	na	-	36/23/22	9/7/4	0.730	43/23/16	2/7/10	0.001
*TP53* WT/mut	na	na	-	65/16	20/0	0.037	82/0	3/16	< 0.001
*SF3B1* WT/mut	na	na	-	73/8	17/3	0.452	74/8	16/3	0.429
*NOTCH1* WT/mut	na	na	-	73/8	17/3	0.452	72/10	18/1	0.685
*ATM* WT/mut	na	na	-	76/5	17/3	0.192	74/8	19/0	0.346
*BIRC3* WT/mut	na	na	-	77/4	19/1	1.000	79/3	17/2	0.236
Others WT/mut	na	na	-	60/21	15/5	1.000	61/21	14/5	1.000

*TP53 disruption: 17p13 deletion by FISH and/or *TP53* mutation by NGS.

^TP53 mutations not included.

Abbreviations: f, female; fav, favorable; int, intermediate; m, male; mut, mutated; na; not assessed; neg, negative; pos, positive; unfav, unfavorable; unmut, unmutated; yrs, years.

**Table 3:** Correlations between clinical biologic parameters and cytogenetic findings

	Karyotype abnormality			Complex karyotype		
variable	Fav-int	Unfav	*P*	No	Yes	*P*
Age < 65/> = 65 years	36/30	19/15	1.000	46/23	9/12	0.227
Sex m/f	41/25	22/12	0.831	51/28	12/9	0.614
Binet stage a/b-c	52/14	23/11	0.234	61/18	14/7	0.396
CD38 neg/pos	26/40	14/20	1.000	33/46	7/14	0.618
FISH fav-int/unfav	65/1	8/26	< 0.001	64/14	8/13	< 0.001
*IGHV* mut/unmut	5/61	3/31	1.000	6/73	2/19	0.673
*TP53* disruption no/yes	57/9	25/9	0.168	69/10	13/8	0.021
Mutations s by NGS no/yes	33/33	12/22	0.204	40/39	5/16	0.047
n. of mutations by NGS no/1/≥ 2	33/16/17	12/14/8	0.201	40/20/19	5/10/6	0.061
*TP53* WT/mut	57/9	28/6	0.572	70/9	15/6	0.080
*SF3B1* WT/mut	59/7	31/3	1.000	72/7	18/3	0.434
*NOTCH1* WT/mut	60/6	29/5	0.502	70/9	19/2	1.000
*ATM* WT/mut	63/3	29/5	0.117	75/4	17/4	0.058
*BIRC3* WT/mut	63/3	32/2	1.000	75/4	20/1	1.000
Others WT/mut	49/17	25/9	1.000	59/20	15/6	0.480

Abbreviations: f, female; fav, favorable; int, intermediate; m, male; mut, mutated; na; not assessed; neg, negative; pos, positive; unfav, unfavorable; unmut, unmutated; yrs, years.

### Correlations between mutational status, cytogenetic findings and clinical and biological parameters

The presence of gene mutations did not correlate with age, sex, Binet stage and CD38 positivity, while a significant association was found with unmutated *IGHV* status (*p* = 0.040) and a complex karyotype (*p* = 0.047; Tables 2 and 3). A complex karyotype (Table 3) was also associated with unfavorable FISH (*p* < 0.001) and *TP53* disruption (*p* = 0.012). A trend for a significant association was observed with the presence of ≥ 2 mutations (*p* = 0.061) and with *TP53* and *ATM* mutations (*p* = 0.080 and 0.058, respectively). A positive correlation was found between unfavorable FISH and unfavorable karyotype (*p* < 0.001). 11q22 deletions by FISH were never found in association with *TP53* mutations or with *TP53* disruption. *TP53* disruption was associated with the presence of ≥ 2 mutations (*p* = 0.001) and mutated *IGHV* status (*p* < 0.001), the latter association being accounted for by the selection criteria adopted in this analysis, which included patients with unmutated *IGHV* and *TP53* lesions. An overview of the distribution of mutations and principal biological findings according to cytogenetic results is presented in Supplementary Figure 1.

### Correlations between mutational status, cytogenetic findings and clinical outcome

**Figure 1:**
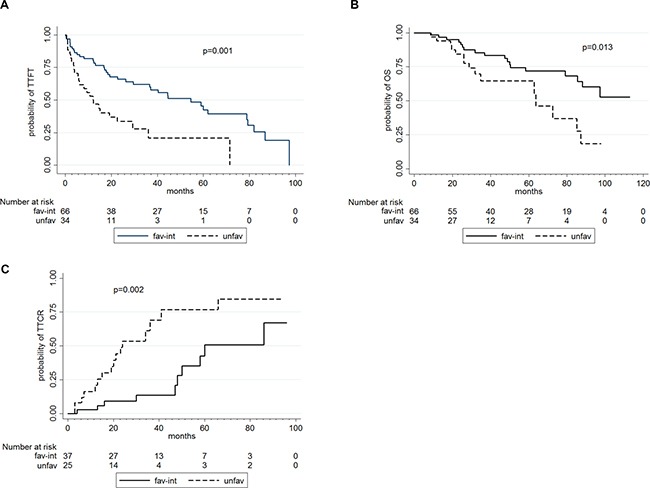
TTFT (in **A**), OS (in **B**) and TTCR (in **C**) according to karyotype abnormalities.

The median follow-up for these 101 CLL patients was 41 months. In univariate analysis (Table 4), a worse TTFT was associated with advanced Binet stage (*p* < 0.001), a complex karyotype (*p* < 0.001) and an unfavorable karyotype (*p* = 0.001; Figure 1A). No correlations were observed between TTFT and gene mutations.

**Table 4:** Univariate analysis for TTFT and OS

		TTFT		OS	
Variable	*N pts*	HR (CI 95%)	*P*	HR (CI 95%)	*P*
Binet stage b-c vs a	*25 vs 76*	2.441 (1.851–3.218)	< 0.001	1.457 (1.021–2.080)	0.038
CD38 pos vs neg	*60 vs 41*	1.490 (0.884–2.512)	0.134	0.931 (0.466–1.859)	0.839
*IGHV* unmut vs mut	*92 vs 9*	2.169 (0.774–6.080)	0.141	1.596 (0.382–6.673)	0.522
11q22 deletion yes vs no	*20 vs 81*	1.350 (0.711–2.561)	0.359	0.946 (0.389–2.302)	0.903
*TP53* disruption yes/no	*19 vs 82*	1.340 (0.733–2.452)	0.342	2.419 (1.145–5.111)	0.021
Complex karyotype yes vs no	*21 vs 79*	3.024 (1.705–5.362)	< 0.001	3.364 (1.596–7.091)	0.001
Mutations by NGS yes/no	*56 vs 45*	0.662 (0.395–1.109)	0.117	0.694 (0.341–1.414)	0.315
Karyotype abnormalities unfav vs fav-int	*34 vs 66*	1.611 (1.228–2.112)	0.001	1.562 (1.099–2.219)	0.013
*TP53* mut vs wt	*16 vs 85*	1.107 (0.571–2.149)	0.763	1.612 (0.697–3.728)	0.264
*SF3B1* mut vs wt	*11 vs 90*	1.120 (0.753–1.666)	0.575	1.210 (0.666–2.195)	0.532
*NOTCH1* mut vs wt	*11 vs 90*	0.739 (0.519–1.050)	0.096	0.744 (0.476–1.164)	0.196
*ATM* mut vs wt	*8 vs 93*	0.716 (0.466–1.100)	0.127	0.810 (0.479–1.370)	0.432
*BIRC3* mut vs wt	*5 vs 96*	1.287 (0.719–2.305)	0.396	1.104 (0.538–2.269)	0.787
OTHERS mut vs wt	*26 vs 75*	0.958 (0.725–1.267)	0.766	1.126 (0.741–1.710)	0.578

A poorer OS (Table 4), was associated with advanced Binet stage (*p* = 0.038), *TP53* disruption (*p* = 0.021), complex karyotype (*p* = 0.001) and an unfavorable karyotype (*p* = 0.013; Figure 1B).

In univariate analysis (Table 5), a shorter TTCR correlated with unmutated *IGHV* status, (*p* = 0.036), *TP53* disruption (*p* < 0.001), a complex karyotype (*p* = 0.004), an unfavorable karyotype (*p* = 0.002; Figure 1C) and *TP53* mutations (*p* = 0.013).

**Table 5:** Univariate analysis for TTCR

		TTCR	
Variable	*N pts*	HR (CI 95%)	*P*
Binet stage b-c vs a	*24 vs 39*	1.159 (0.783–1.715)	0.463
CD38 pos vs neg	*40 vs 23*	0.840 (0.370–1.904)	0.676
*IGHV* unmut vs mut	*58 vs 5*	0.148 (0.037–0.899)	0.036
11q22 deletion yes vs no	*12 vs 51*	1.409 (0.590–3.364)	0.441
*TP53* disruption yes/no	*15 vs 48*	6.021 (2.366–15.320)	< 0.001
Complex karyotype yes vs no	*18 vs 44*	3.153 (1.429–6.954)	0.004
Mutations by NGS yes/no	*40 vs 23*	0.717 (0.311–1.653)	0.435
Karyotype abnormalities unfav vs fav-int	*25 vs 37*	1.864 (1.250–2.779)	0.002
*TP53* mut vs wt	*12 vs 51*	3.484 (1.297–9.355)	0.013
*SF3B1* mut vs wt	*7 vs 56*	1.159 (0.271–4.952)	0.842
*NOTCH1* mut vs wt	*9 vs 54*	1.222 (0.419–3.561)	0.713
*ATM* mut vs wt	*6 vs 57*	1.107 (0.328–3.734)	0.870
*BIRC3* mut vs wt	*3 vs 60*	1.144 (0.263–4.982)	0.858
OTHERS mut vs wt	*18 vs 45*	0.696 (0.309–1.566)	0.381
Lines of therapy 1 vs > 1	*37 vs 26*	1.062 (0.437–2.580)	0.894

By multivariate analysis (Table 6) an advanced Binet stage (*p* < 0.001) and an unfavorable karyotype (*p* = 0.001) predicted a shorter TTFT while *TP53* disruption (*p* = 0.019) and an unfavorable karyotype (*p* = 0.028) were associated with a worse OS. A shorter TTCR (Table 6) was predicted, in multivariate analysis, by *TP53* disruption (*p* = 0.001) and unfavorable cytogenetics (*p* = 0.025).

**Table 6:** Multivariate analysis for TTFT, OS and TTCR

	Variable				After bootstrapping	
TTFT		HR	CI	*p*	CI	*p*
	Binet stage b-c vs a	2.457	1.848–3.264	< 0.001	1.788–3.374	< 0.001
	Karyotype abnormalities unfav vs fav-int	1.630	1.231–2.159	0.001	1.241–2.141	< 0.001
**OS**						
	Binet stage b-c vs a	1.414	0.978–2.049	0.065	0.891–2.246	0.142
	*TP53* disruption yes vs no	3.029	1.406–6.527	0.005	1.200–7.648	0.019
	Karyotype abnormalities unfav vs fav-int	1.512	1.046–2.184	0.028	1.031–2.218	0.034
**TTCR**						
	*IGHV* unmut vs mut	0.583	0.113–3.018	0.522	0.321–8.060	0.960
	*TP53* disruption yes vs no	5.049	1.820–14.009	0.002	1.894–13.458	0.001
	Karyotype abnormalities unfav vs fav-int	1.761	1.171–2.649	0.007	1.072–2.894	0.025

The following variables were included in multivariate analysis:

TTFT: Binet stage and karyotype abnormalities.

OS: Binet stage, *TP53* disruption and karyotype abnormalities.

TTCR: *IGHV*, *TP53* disruption and karyotype abnormalities.

When in the model of multivariate analysis we introduced the complex karyotype instead of karyotypic abnormalities, the complex karyotype was significantly associated with a worse TTFT (*p* < 0.001) and an inferior OS (*p* = 0.024) while a trend for significance was observed for a shorter TTCR (*p* = 0.077; Supplementary Table 4).

**Figure 2:**
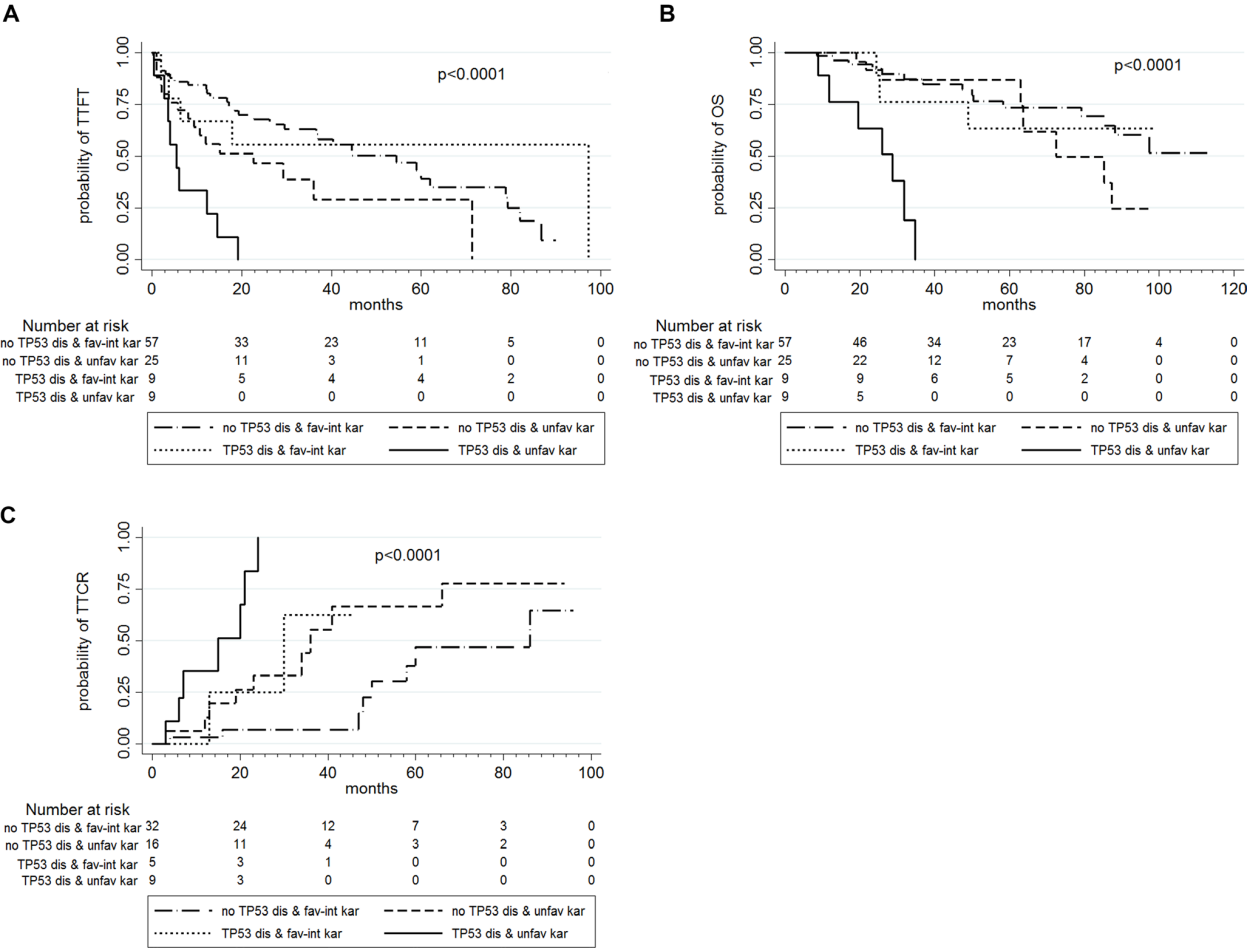
TTFT (in **A**), OS (in **B**) and TTCR (in **C**) according to *TP53* disruption status and karyotype abnormalities.

By combining *TP53* disruption status and cytogenetic results, 9 patients showed the coexistence of *TP53* disruption and unfavorable karyotype: this subset of very high-risk patients presented a median TTFT of 5.5 months, a median OS of 28.7 months and a median TTCR of 15 months (Table 7, Figure 2).

**Table 7:** Median TTFT, OS and TTCR according to TP53 disruption status and karyotype abnormalities

	n. pts	TTFT*		OS*		N pts	TTCR*	
		Median months	se	Median months	se		Median months	se
No *TP53* disruption & fav-int karyotype	57	54.5	11.1	NR	-	32	86.0	17.4
*TP53* disruption & fav-int karyotype	9	97.3	1.3	NR	-	5	30.0	6.1
No *TP53* disruption & unfav karyotype	25	22.6	9.2	72.5	14.5	16	36.0	2.7
*TP53* disruption & unfav karyotype	9	5.5	2.2	28.7	6.2	9	15.0	5.2

*P < 0.0001 for the comparison by log-rank test.

Abbreviations: fav: favorable; int: intermediate; NR: not reached; se: standard error; unfav: unfavorable.

In patients with favorable or intermediate cytogenetic findings (Supplementary Table 5), *TP53* mutations correlated with a shorter TTCR (*p* = 0.037; Supplementary Figure 2) while in patients without *TP53* disruption or complex karyotype (Supplementary Table 6), NOTCH1 mutations predicted a shorter TTFT and an inferior OS (*p* = 0.008 and 0.016, respectively; Supplementary Figures 3 and 4). A worse TTFT was also associated with the presence of ≥ 2 gene mutations (*p* = 0.038).

## DISCUSSION

*IGHV* status and FISH results represent robust prognostic tests and their inclusion in the standard prognostic work up of newly diagnosed and/or previously untreated CLL was recently recommended [25]. Indeed unmutated *IGHV* status and 11q22 deletion were recognized as independent predictors of an unfavorable outcome [23, 24] while 17p13 deletion was aggregated with *TP53* mutations in a category that received the highest risk score in the international prognostic index (IPI) of CLL [24]. Thus, based on *IGHV* status, FISH results and *TP53* mutational analysis it is possible to identify a category of CLL patients with poor prognosis, representing approximately 50% of all CLL cases [13].

Karyotype analysis was reinforced as an important variable in CLL risk assessment [29, 31, 32, 33] though it is relatively time consuming and needs standardization of culture conditions [34]. Because few data are available on cytogenetic and molecular findings in high-risk CLL, we perform an extensive genetic characterization of 101 CLL with unmutated *IGHV* and/or 11q22 deletion and/or *TP53* disruption. These patients were diagnosed and followed at a single center for a median of 41 months over the last 10 years. Notably, our center has a > 90% capture of incident CLL cases in our region allowing for meaningful analyses of TTFT, TTCR and OS in a real world scenario.

In this study, cytogenetic analysis was performed following DSP30/IL2 stimulation, thus reducing the rate of cytogenetic failure [28, 34]. A complex karyotype was present at diagnosis in 20.8% of patients, while a favorable karyotype was observed in 29.7% of the cases. Similar data were observed in the CLL 11 trial when karyotyping was performed before treatment and chromosomal aberrations were found in 68.8% of 154 patients, with 19.5% of the cases showing a complex karyotype [30]. These figures, as expected, are higher than those observed in unselected series of patients that included also low-risk CLL and cases with stable disease [29, 33].

We performed mutational screening with the Ion Torrent PGM, a very sensitive NGS platform, allowing for multiplexing of samples and gene targets in one experimental setup thus resulting in higher speed of analysis and lower costs [35]. Parallel sequencing of exonic regions in 20 CLL-related genes showed gene mutations in 56/101 (55.4%) cases by using a 5% cut off. Mutations were detected with a frequency ranging from 5.0 to 96.7% of the reads, showing cases with major clones that represent early leukemogenetic events and cases with minor clones that represent late-appearing aberrations [36, 37]. In this series, the frequency of mutations involving *TP53*, *NOTCH1*, *SF3B1*, *ATM* and *BIRC3* genes (15.8, 10.9%, 10.9%, 7.9% and 4.9%, respectively) reflected the high-risk genetic features of our patients. Similar incidences for these mutations were reported in patients enrolled in clinical trials at disease progression [13]. The frequency of mutations involving the other investigated genes was in line with published data using whole exome sequencing [38–41]. Interestingly, we observed that 25.7% of the cases presented more than one mutation. In the CLL11 trial, NGS analysis revealed mutations in 42 out of 85 analyzed genes, with 76.4% and 42.2% of the patients presenting one or ≥ 2 mutations, respectively [30].

Moreover, an unfavorable karyotype, defined by the presence of a complex karyotype and/or 11q22 and/or 17p13 deletion, was independently associated with a worse OS and TTFT, while *TP53* disruption was associated with poorer OS. It is worth noting, however, that our patients may have received different lines of treatment including various chemotherapeutic agents, with or without rituximab and BTK inhibitors. These observations need therefore to be validated in larger series of patients and in clinical trials with homogeneous treatments. Interestingly, in the CLL11 trial a complex karyotype emerged as an independent negative prognostic factor for OS after front-line therapy [30] while in relapsed/refractory CLL treated with ibrutinib-based regimens, a complex karyotype was stronger than 17p13 deletion in predicting an inferior outcome [31]. A complex karyotype was also found to be associated with unmutated *IGHV* genes and aberrations of chromosome 17p and was identified as an independent prognostic factor for shorter TTFT in a series of 1001 cases [33]. Likewise, an unfavorable karyotype predicted a shorter OS and represented the strongest prognostic parameter for disease progression in unselected patients studied at diagnosis [28, 29].

The mechanisms responsible for the unfavorable prognostic significance of the complex karyotype remain to be investigated, though in CLL it was shown that telomere shortening was associated with genetic complexity [42].

We also assessed the impact of karyotyping and gene mutations on TTCR. While confirming the negative role of *TP53* disruption [36, 37], we showed, for the first time, that patients with unfavorable cytogenetics had a shorter TTCR. The identification of patients that are likely to develop chemoresistance is very important, given the possibility to use alternative effective BCR-target treatments [43, 44].

Nor the presence of gene mutations, nor single gene mutations correlated with TTFT, OS and TTCR, with the notable exception of *TP53* mutations. This is not surprising if we consider that the high-risk genetic profile of our patients may mirror a genetic instability or dysregulation [45] that may overcome the prognostic impact of single gene mutations [12, 13].

Interestingly our data showed that high-risk CLL patients can be further sub-classified into four different groups, based on *TP53* disruption status and karyotype. Those patients with both unfavorable cytogenetics and *TP53* disruption presented a dismal clinical course (median TTFT, OS and TTCR of 5.5, 28.7 and 15.0 months, respectively). This observation may justify a specific treatment strategy for this subset of patients [46]. By contrast, there are cases with *TP53* disruption and favorable or intermediate cytogenetics that may display a less aggressive course of the disease [47]. Recently, it has been shown that also the percentage of positive cells by FISH may help to refine the prognosis of high-risk CLL patients [48]. By contrast, mutational analysis may refine the prognosis of high-risk patients without unfavorable or complex karyotype or *TP53* disruption as shown by the negative prognostic impact of *NOTCH1* mutations and of the coexistence of two or more mutations [49].

In conclusion, we described the results of a comprehensive analysis of chromosomal aberrations and gene mutations in high-risk CLL, providing the first demonstration that the cytogenetic profile was independently associated with a shorter TTFT, OS and TTCR. The introduction of this technique in future CLL trials seems warranted to identify those high-risk patients that could be considered as ideal candidates for consolidation treatments or novel treatment combinations [50–53]. Larger series of homogenously treated patients and with longer follow-up could confirm this observation.

## MATERIALS AND METHODS

### Patients

One hundred-one untreated CLL patients with unmutated *IGHV* gene and/or 17p13/11q22 deletion by FISH and/or mutated *TP53* gene (here referred to as high-risk CLL) were included in this analysis. *TP53* disruption was defined by the presence of 17p13 deletion by FISH and/or *TP53* mutation by NGS. These patients belong to a consecutive series of 200 patients diagnosed and followed between 2007 and 2014 [49]. All patients were diagnosed according to NCI criteria [54]. Only patients with a Matutes immunophenotypic score [55] ≥ 3 (i.e., typical CLL) were included. CD38 and ZAP70 were tested on peripheral blood (PB) cells, as described [56]. When needed, mantle cell lymphoma was excluded by cyclin D1 evaluation. The study was approved by the local ethics committee. Indications for treatment included: increased white blood cell count with < 6 month lymphocyte doubling time, anemia or thrombocytopenia due to bone marrow infiltration or autoimmune phenomena not responding to steroids, and clinically significant disease progression in the Binet staging system. Fludarabine or bendamustine containing regimens, with or without rituximab were used as first-line treatment in fit patients; chlorambucil with or without rituximab was used in elderly and/or unfit patients according to the treatment policy adopted at our center. Since 2015, ibrutinib or idelalisib plus rituximab were offered to relapsed/refractory patients.

### Cytogenetic and FISH analyses

Interphase FISH was performed on PB samples obtained at diagnosis using probes for the following regions: 13q14, 12q13, 11q22/ATM, 17p13/TP53 (Vysis/Abbott Co, Downers Grove, IL) as described [57]. Each patient was categorized into a FISH risk group according to the following classification: favorable group (isolated 13q14 deletion or absence of FISH aberrations), intermediate group (trisomy 12); unfavorable group (deletions of 11q22 or of 17p13).

Cytogenetic analysis was performed on the same samples used for FISH analysis using CpG-oligonucleotide DSP30 (2 μmol/l TibMolBiol Berlin, Germany) plus IL2 (100 U/ml Stem Cell Technologies Inc., Milan, Italy) as described [57]. Whenever possible, at least 20 metaphases were karyotyped and each patient was categorized into a cytogenetic risk group according to the following classification: favorable group (isolated 13q14 deletion or normal karyotype), unfavorable group (deletions of 11q22 or 17p13, or complex karyotype, ie, at least three chromosome aberrations); intermediate group (all other karyotypic abnormalities).

### IGHV analysis

*IGHV* genes were amplified from genomic DNA and sequenced according to standard methods with the cut-off of 98% homology to the germline sequence to discriminate between mutated (< 98%) and unmutated (≥ 98%) cases, as reported [58].

### Next generation sequencing

NGS analysis was performed on the same samples used for FISH and cytogenetic analyses. In all samples, the percentage of CLL cells was over 90% as assessed by flow cytometry analysis. Agilent HaloPlex Target Enrichment kit (Agilent Technologies, Santa Clara, CA, USA) was used to produce libraries of exonic regions from 20 genes (*ATM, BIRC3, BRAF, CDKN2A, PTEN, CDH2, DDX3X, FBXW7, KIT, KLHL6, KRAS, MYD88, NOTCH1, NRAS, PIK3CA, POT1, SF3B1, TP53, XPO1, ZMYM3*) starting from genomic DNA from PB samples, according to HaloPlex Target Enrichment System (Agilent Technologies, Santa Clara, CA, USA). Diluted libraries were linked to Ion Sphere Particles, clonally amplified in an emulsion PCR and enriched using Ion OneTouch emulsion PCR System (Life technologies, Foster City, CA, USA). Exon-enriched DNA was precipitated with magnetic beads coated with streptavidin. Enriched, template-positive Ion Sphere Particles were loaded in one Ion chip and sequenced using Ion Torrent PGM (Life technologies, Foster City, CA, USA). Sequencing data were aligned to the human reference genome (GRCh37). Data analysis and variants identification were performed using Torrent Suite 3.4 and Variant Caller plugin 3.4.4 (Life technologies, Foster City, CA, USA). To identify pathogenic variations, mutations that did not affect the protein coding regions (intronic, 3′ and 5′ UTR variations, silent exonic mutations and polymorphisms) were filtered out; insertions and deletions belonging to homopolymeric regions were removed, because sequencing error rate is high in these regions [49].

### Statistical analysis

The Fischer's exact test was applied for categorical variables. TTFT was calculated as the interval between diagnosis and the start of first line treatment. OS was calculated from the date of diagnosis until death due to any cause or until the last patient follow-up. Refractory disease was defined as treatment failure (stable disease, nonresponse, progressive disease, or death from any cause) or disease progression within 6 months from antileukemic therapy [54]. TTCR was measured from date of first-line treatment until date of refractoriness to fludarabine/bendamustine-based regimens, date of alkylator refractoriness in patients who had never been exposed to fludarabine/bendamustine, death, or last follow-up [59]. Survival curves were compared by the log-rank test. Proportional hazards regression analysis was used to identify the significant independent prognostic variables on TTFT. The stability of the Cox model was internally validated using bootstrapping procedures [11]. Statistical analysis was performed using Stata 14.0 (Stata Corp, College Station, TX).

## SUPPLEMENTARY MATERIALS FIGURES AND TABLES






