# Supplementary material for: An extensive molecular cytogenetic characterization in high-risk chronic lymphocytic leukemia identifies karyotype aberrations and TP53 disruption as predictors of outcome and chemorefractoriness
Source: Oncotarget. 2017 Mar 3;8(17):28008–20. doi: 10.18632/oncotarget.15883 (PMC5438626; doi:10.18632/oncotarget.15883)
Supplement: Supplementary file 1 [file oncotarget-08-28008-s001.pdf]

An extensive molecular cytogenetic characterization in high-risk chronic lymphocytic leukemia identifies karyotype aberrations and *TP53* disruption as predictors of outcome and chemorefractoriness

Supplementary Materials

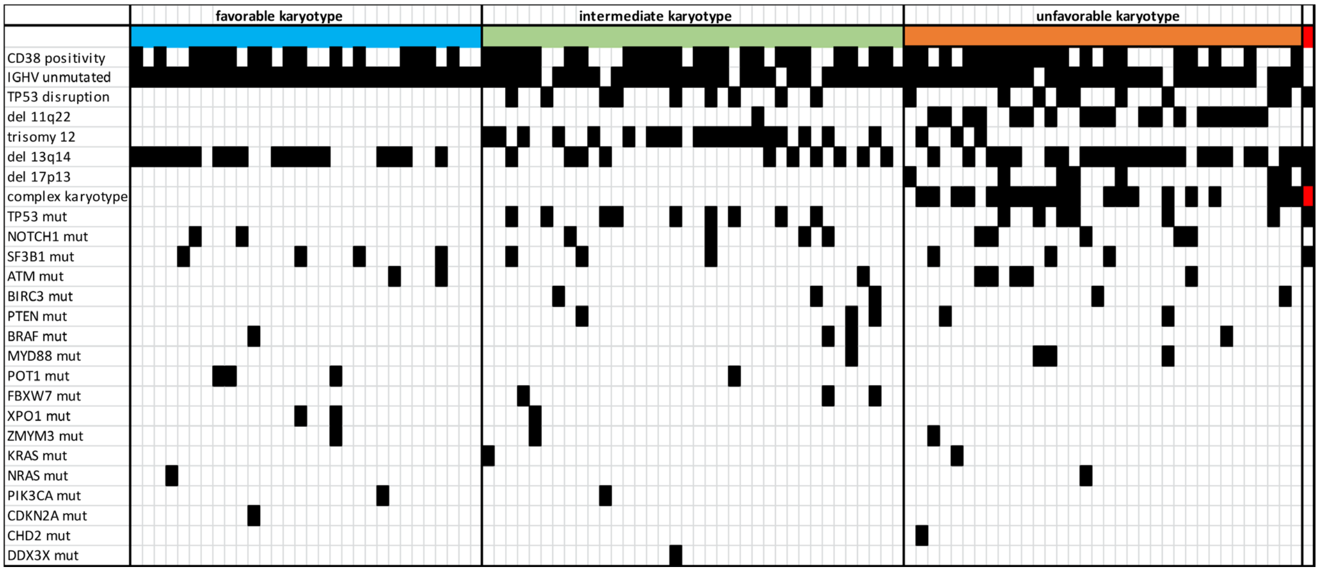

Supplementary Figure 1: Clustering diagram showing NGS results and principal biological findings according to cytogenetic results (favorable, intermediate and unfavorable karyotype). In one case (indicated with the red color) cytogenetic analysis was not assessable.

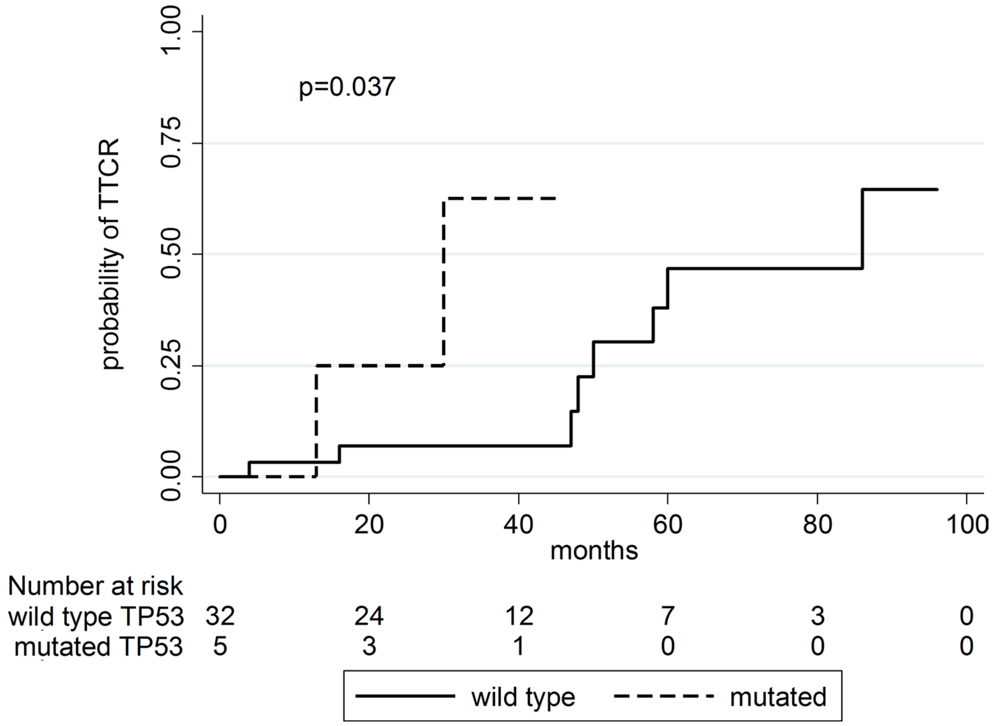

Supplementary Figure 2: TTCR according to *TP53* status in patients with favorable or intermediate karyotype.

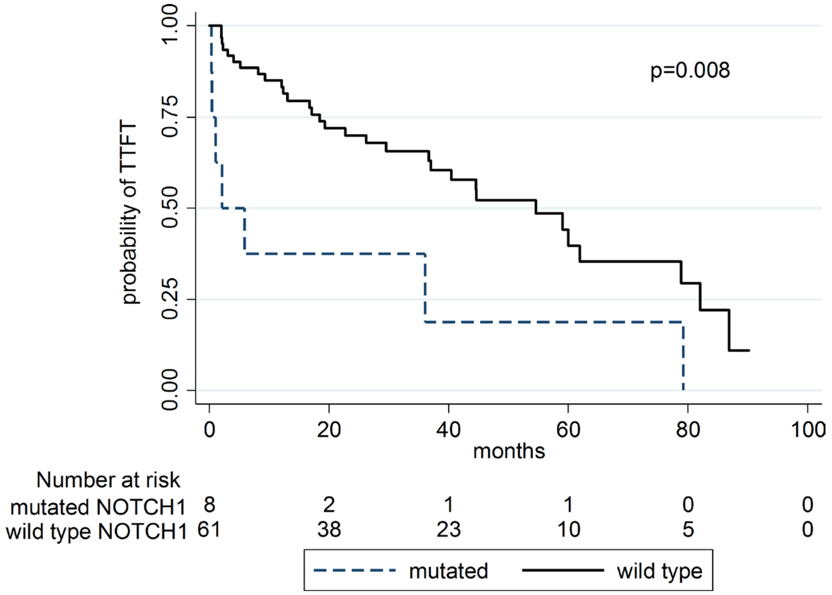

Supplementary Figure 3: TTFT according to *NOTCH1* status in patients without *TP53* disruption or complex karyotype.

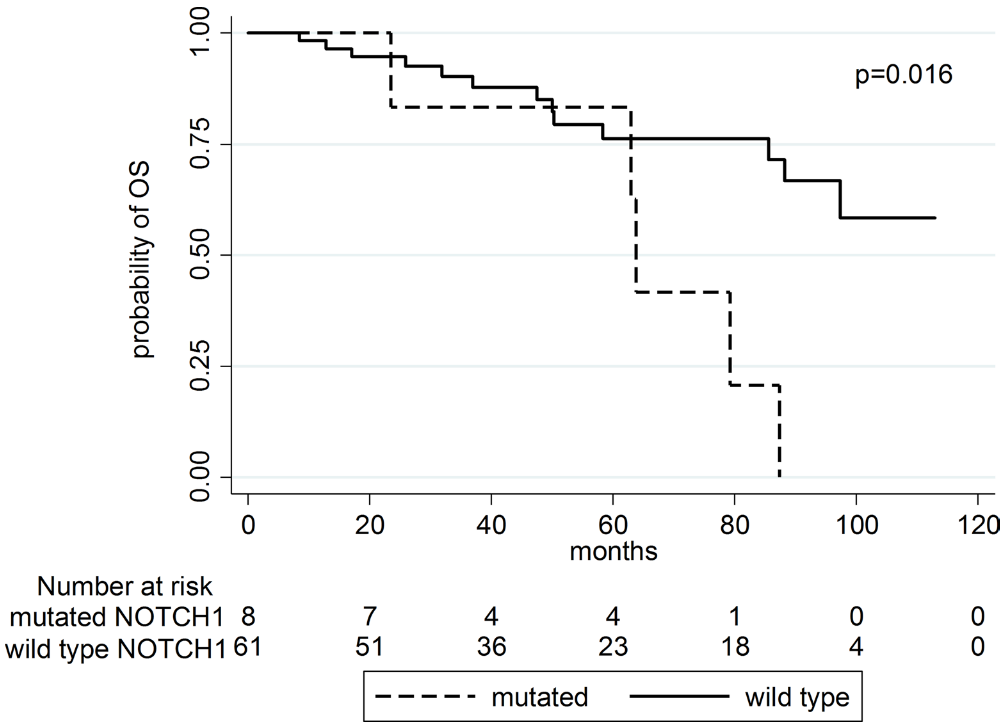

Supplementary Figure 4: OS according to *NOTCH1* status in patients without *TP53* disruption or complex karyotype.

Supplementary Table 1: List of karyotypes in the 101 high-risk CLL patients. See Supplementary_ Table_1.

Supplementary Table 2: Overview of the mutations observed in the 20 genes and median coverage for each gene

Gene	Total mutated cases	Median coverage
TP53	16	871.8
NOTCH1	11	708.4
SF3B1	11	719.4
ATM	8	566.4
BIRC3	5	466.2
PTEN	5	498.3
BRAF	4	495.0
MYD88	4	683.5
POT1	4	524.0
FBXW7	3	781.4
XPO1	3	545.3
ZMYM3	3	519.8
KRAS	2	664.6
NRAS	2	732.6
PIK3CA	2	954.9
CDKN2A	1	531.0
CHD2	1	653.6
DDX3X	1	712.0
KIT	0	650.0
KLHL6	0	1265.3

Supplementary Table 3: List of gene mutations by NGS in the 56 mutated high-risk CLL patients.
See Supplementary_Table_3.

Supplementary Table 4: Multivariate analysis for TTFT, OS and TTCR with complex karyotype instead of karyotype abnormalities

Variable					After bootstrapping	
**TTFT**		**HR**	**CI**	***p***	CI	***p***
	Binet stage b-c vs a	2.410	1.818–3.195	< 0.001	1.845–3.147	< 0.001
	Complex karyotype	2.934	1.625–5.298	< 0.001	1.686–5.108	< 0.001
**OS**						
	Binet stage b-c vs a	1.451	1.006–2.094	0.046	0.916–2.300	0.113
	TP53 disruption yes vs no	2.825	1.296–6.258	0.009	1.148–6.951	0.035
	Complex karyotype	2.914	1.357–6.258	0.006	1.080–7.861	0.024
**TTCR**						
	IGHV unmut vs mut	0.632	0.121–3.297	0.586	0.174–2.300	0.971
	TP53 disruption yes vs no	4.771	1.729–13.163	0.003	1.157–19.360	0.029
	Complex karyotype	2.486	1.086–5.685	0.031	0.905–6.825	0.077

The following variables were included in multivariate analysis:
TTFT: Binet stage and complex karyotype.
OS: Binet stage, *TP53* disruption and complex karyotype.
TTCR: *IGHV*, *TP53* disruption and complex karyotype.

Supplementary Table 5: Prognostic impact of mutations in patients with favorable or intermediate karyotype

		TTFT			OS			TTCR		
**Variable**	***N pts****	**HR (CI 95%)**	***P***	***p***	**HR (CI 95%)**	***P***	***p***	**HR (CI 95%)**	***P***	***p***
Mutations by NGS yes/no	*33 vs 33 [21 vs 16]*	1.384 (0.717–2.675)	0.333	-	1.375 (0.540–3.496)	0.504	-	1.169 (0.327–4.170)	0.810	--
Number of mutations >=2 vs 1 vs 0	*17 vs 16 vs33 [11 vs 10 vs 16]*	1.344** (0.609–2.967)<br>1.427** (0.646–3.151)	0.463<br>0.379	0.618	1.339** (0.436–4.116)<br>1.411** (0.458–4.345)	0.609<br>0.548	0.796	0.778** (0.142–4.280)<br>1.557** (0.387–6.271)	0.773<br>0.553	0.693
TP53 mut vs wt	*9 vs 57 [5 vs 32]*	0.605 (0.210–1.743)	0.351	-	1.170 (0.339–4.051)	0.805	-	8.065 (1.133–57.394)	0.037	-
SF3B1 mut vs wt	*7 vs 59 [na]*	0.571 (0.374–3.050)	0.902	-	0.981 (0.224–4.286)	0.979	-	Na		-
NOTCH1 mut vs wt	*6 vs 60 [na]*	1.630 (0.571–4.657)	0.362	-	0.682 (0.323–1.440)	0.316	-	Na		-

*In squared brackets patients for TTCR analysis.
**versus no mutation.
Legend: f, female; fav, favorable; HR: hazard ratio; int, intermediate; m, male; mut, mutated; na; not assessed; neg, negative; OS: overall survival; pos: positive; TTCR: time to chemorefractoriness; TTFT: time to first treatment; unfav, unfavorable; unmut, unmutated; yrs, years.

Supplementary Table 6: Prognostic impact of mutations in patients without *TP53* disruption or complex karyotype

		TTFT			OS			TTCR		
**Variable**	***N pts****	**HR (CI 95%)**	***p***	***p***	**HR (CI 95%)**	***p***	***p***	**HR (CI 95%)**	***p***	***p***
Mutations by NGS Yes vs no	*30 vs 39 [20 vs 18]*	1.887 (0.985–3.618)	0.056	-	0.843 (0.699–4.503)	0.228	-	1.732 (0.517–5.795)	0.373	-
pNumber of mutations >=2 vs 1 vs 0	*12 vs 18 vs 39 [9 vs 11 vs 18]*	1.619** (0.754–3.475) 2.362** (1.047–5.329)	0.216 0.038	0.092	1.831** (0.632–5.308) 1.039** (0.509–5.638)	0.265 0.390	0.469	0.580** (0.144–2.330) 1.009** (0.251–4.058)	0.443 0.990	0.666
SF3B1 mut vs wt	*5 vs 64 [na]*	0.999 (0.304–3.277)	0.998	-	0.632 (0.084–4.761)	0.656	-	Na		-
NOTCH1 mut vs wt	*8 vs 61 [7 vs 31]*	3.113 (1.352–7.169)	0.008	-	3.722 (1.284–10.798)	0.016	-	0.581 (0.152–2.227)	0.429	-

*In squared brackets patients for TTCR analysis.

**versus no mutation.

Legend: f, female; fav, favorable; HR: hazard ratio; int, intermediate; m, male; mut, mutated; na; not assessed; neg, negative; OS: overall survival; pos: positive; TTCR: time to chemorefractoriness; TTFT: time to first treatment; unfav, unfavorable; unmut, unmutated; yrs, years.
