# Supplementary material for: An extensive molecular cytogenetic characterization in high-risk chronic lymphocytic leukemia identifies karyotype aberrations and TP53 disruption as predictors of outcome and chemorefractoriness
Source: Oncotarget. 2017 Mar 3;8(17):28008–20. doi: 10.18632/oncotarget.15883 (PMC5438626; doi:10.18632/oncotarget.15883)
Supplement: Supplementary file 2 [file oncotarget-08-28008-s002.docx]

| **sample** | **FISH** | **karyotype** |
| --- | --- | --- |
| 123 | Del13q | 46,XY,t(13;22)(q14;q12)[6]/46,XY[14] |
| 194 | Del17p | 45_~_46,XX,der(2)(p23),add(16)(p13),del(14)(q32),der(17)(p12),+19,-21[cp10]/46,XX[10] |
| 288 | Del13q, trisomy 12 | 47,XX,+12[16]/46XX[4] |
| 431 | Del11q trisomy 12 | 47,XY,del(11)(q11),+12[13]/46,XY[7] |
| 435 | Del11q, del13q | 46,XY,t(1;13)(p32;q14),del(13)(q13q32)[6]/46,idem,del(11)(q14),del(13)(q13q32)[11]/46,XY[3] |
| 441 | Del13q | 46,XY,del(14)(q32)[2]/46,XY[18] |
| 453 | Del13q | 46,XY,del(14)(q31q32)[3]/46,XY[17] |
| 455 | Trisomy 12 | 47,XY,+12[7]/47,idem,add(19)(p13)[3]/46,XY[10] |
| 654 | Del13q, del17p | 46,XX,del(13)(q14q22),del(17)(p12)[6]/46,idem,del(6)(q21)[6]/46,XX[8] |
| 656 | Trisomy 12 | 47,XX,+12[17]/46,XX[3] |
| 660 | Del11q, del13 | 46,XX,del(13)(q14q22)[2]/46,idem,add(2)(q36),t(4;?11)(q22;?q21)[15]/ 46,XX[3] |
| 674 | Del17p | 42_~_45,XX,del(1)(p35),der(4)t(4;?11)(p16;?q13),del(7)(q22),add(8)(p23),-9,add(17)(q25),-17,+5mar[cp12][12]/43-45,XX,del(1)(p35),t(3;5)(q12;p14),der(4)t(4;?11)(p16;?q13),add(8)(p23),add(9)(p24),-14,-17,+dic(19;22)(p13),+2mar[cp7][7] /46,XX[1]45,XX,del(1)(p35),t(3;5)(q12;p14),der(4)t(4;?11)(p16;?q13),add(8)(p23),add(9)(p24),-14,-17,+dic(19;22)(p13),+mar,+mar1 [cp7] [7] / 46,XX [1] |
| 676 | Del11q, del13q | 48,XX,der(1)add(1)(p36)del(1)(q25),add(2)(q23),t(2;?14)(p25;?q22),-3,+6,+12,del(13)(q14q22),+14[19]/46,XX[1] |
| 678 | Trisomy 12 | 47,XY,+12[4]/46,XY[16] |
| 680 | Del17p | 46,XY,del(17)(p13)[6]/46,XY[14] |
| 692 | Normal | 46,XX[20] |
| 694 | Trisomy 12 | 47,XY,+12[6]/46,XY[14] |
| 876 | Del11q, del13q | 46,XY,del(11)(q21),t(13;14)(q14;q24) [8]/46,XY,del(11)(q21),t(13;14)(q14;q24),del(13)(q14q22)[5]/46,XY[4] |
| 880 | Del13q | 46,XY,del(13)(q14q22)[3]/46,XY[17] |
| 886 | Del13q | 46,XY,del(13)(q14q22)[4]/46,XY[16] |
| 896 | Normal | 46,XY[20] |
| 900 | Del13q | 46,XY,i(8)(q10),t(11;14)(q13;q32),del(11)(q14)[8]/46,idem,del(13)(q14)[2]/49,idem,+5,+12,+22[8]/46,XY[2] |
| 940 | Del13q | 46,XX,del(13)(q14q21)[3]/46,XX[17] |
| 942 | Normal | 47,XY,+del(4)(q11),del(6)(q22)[8]46,XY[15] |
| 964 | Normal | 46,XY,add(2)(q36)[8]/46,idem,del(14)(q24)[5]/46,XY[7] |
| 966 | Normal | 46,XX[20] |
| 974 | Normal | 46,XY[20] |
| 978 | Normal | 46,XY,del(13)(q14q22)[11]/46,XY[10] |
| 1002 | Normal | 40_~_47,XY,del(1)(p11),+del(1)(q11),add(2)(p25),+4,-6,+3mar1[cp15]/46,XY[5] |
| 1006 | Normal | 46,XY[20] |
| 1192 | Trisomy 12 | 47,XX,+12[18]/46,XX[2] |
| 1196 | Trisomy 12 | 47,XX,+12[13]/46,XX[7] |
| 1206 | Trisomy 12 | 47,XX,+12[13]/46,XX[7] |
| 1212 | Del13q | 46,XY,del(13)(q14q22)[6]/46,XY[16] |
| 1222 | Trisomy 12 | 47,XX,+12[20] |
| 1313 | Trisomy 12 | 47,XY,i(8)(q10),+12[12]/47,XY,add(12)(p13)[1]/46,XY[3] |
| 1315 | Normal | 46,XY[20] |
| 1319 | Del13q | 46,XX,del(7)(q22q32)[2]/46,XX,t(13;14)(q14q32)[11]/46,XX[17] |
| 1321 | Del11q, del13q | 46,XX,del(13)(q14q22)[3]/46,XX,del(11)(q11)[2]/46,XX,add(4)(p16),del(11)(q11),del(13)(q14q22)[2]/46,XX[13] |
| 1325 | Normal | 46,XX[20] |
| 1345 | Del11q, del13q | 46,XY,del(11)(q13)[11]/46,XY,idem,del(13)(q14q21)[5]/46,XY[4] |
| 1449 | Del11q, del13q | 46,XX,del(13)(q14q22)[2]/46,idem,del(10)(q23),[3]/46,idem,del(11)(q21)[5] |
| 1451 | Trisomy 12 | 47,XY,+12[20] |
| 1459 | Del11q, del13q | 46,XY,del(13)(q14q22) [6]/46,idem,del(11)(q21)[9]/46,XY[3] |
| 1473 | Normal | 46,XY[20] |
| 1481 | Del13q | 46,XY,t(16;18)(q24;q21)[19]/46,XY[1] |
| 1499 | Normal | 46,XY[20] |
| 1568 | Del11q, trisomy12 | 47,XX,+12[3]/46,XX[17] |
| 1572 | Normal | 46,XY,del(7)(q32)[4]/46,XY[21] |
| 1668 | Del13d, del17p | 44,XY,-5,del(6)(q11),-8,der(10)t(10;?21)(q22;?q21),del(11)(q11),del(13)(q14),del(14)(q24),+der(14)t(5;14)(q21;q32),add(17)(p11),-21[13]/46,XY[5] |
| 1674 | Del13q | 46,XX,del(13)(q14q22)[2]/46,XX[18] |
| 1684 | Trisomy 12 | 47,XY,+12[5]/47,idem,del(14)(q22)[4]/47,idem,t(4;13)(p16;q14)[5]/46,XY [6] |
| 1690 | Del13q | 46,XY,del(13)(q14q22)[5]/46,XY[15] |
| 1835 | Del11q, del13q | 46,XY,del(13)(q14q22)[4]/46,XY,del(11)(q21q22)[3]746,XY[16] |
| 1843 | Trisomy 12 | 47,XY,+12[15]/46,XY[5] |
| 1897 | Del13q | 46,XX,del(13)(q14q22)[5]/46,XX[15] |
| 1901 | Del13q | 43,XY,add(2)(p25),-8,add(11)(p15),der(13)t(13;15)(p13q15),-13,der(14)t(13;14)(q14;p13),-15,der(16)t(8;16)(q12;p13)[cp19]/46,XY[1] |
| 1905 | Trisomy 12 | 47,XX,t(2;18)(p12q21),+12[12]/46,XX[8] |
| 1934 | Del13q. del17p | 44,XY,add(12)(q24),del(13)(q14q32),-16,add(17)(p11),add(18)(p11),-21[15] |
| 1936 | Normal | 46,XY[20] |
| 1938 | Del13q | 46,XY,t(13;16)(q14;q24)[20] |
| 2055 | Del11q, del13q | 46,XY,del(13)(q14q22)[7]/46,idem,del(11)(q21q22)[6]/46,XY[7] |
| 2057 | Del13q | 46,XY,del(13)(q14q22)[9]/46,XY[11] |
| 2117 | Del11q, del13q | 46,XY,del13)(q12q14)[5]/46,idem,del(11)(q14q22)[6]/46,XY[9] |
| 2125 | Del11q | 46,XX,del(11)(q14q22)[18]/46,XX[2] |
| 2131 | Del13q | 46,XY,del(13)(q14q22)[14]/46,XY[6] |
| 2133 | Trisomy 12 | 47,XX,+12[15]/46,XX[5] |
| 2239 | Del11q, del13q | 46,XY,del(11)(q14q22),del(13)(q14q22)[6]/46,XY[24] |
| 2266 | Del11q, del13q | 46,XX,del(11)(q14q22),del(13)(q14q22)[17]/46,XX[3] |
| 2356 | Del13q | 47,XXX,-6,+8[3]/46,XX[16] |
| 2468 | Normal | 46,XY,t(14;18)(q32;q21)[6]/46,XY[14] |
| 2472 | Del11q | 46,XY,+add(X)(p22),-4,add(10)(q25),del(11)(q21),add(17)(p13)[9]/46,XY,del(11)(q21)[8]/46,XY[3] |
| 2476 | Del13q | 46,XX,del(13)(q12q14)[7]/46,XX[13] |
| 2478 | Del13q | 46,XY[20] |
| 2690 | Del13q, del17p | na |
| 2823 | Normal | 45,X,-Y,t(1;15)(p36;q24)[18]/46,XY[2] |
| 3015 | Del13q | 46,XX,del(13)(q14q22)[10]/46,XX[10] |
| 3027 | Normal | 46,XX[20] |
| 3211 | Del13q | 46,XY,del(13)(q14q22)[6]/46,XY[14] |
| 3307 | Trisomy 12 | 47,XY,+12[16]/47,idem,+add(8)(p23),-18[3]/46,XY[1] |
| 3319 | Del13q, del17p | 45,XX,-6,del(13)(q12q14),del(14)(q32),der(17)t(6;17)(p23;p12)[8]/46,XX[12] |
| 3426 | Del13q | 46,XY,del(13)(q14q22)[12]/46,XY[8] |
| 3438 | Del13q | 46,XX,del(13)(q14q22),del(14)(q32)[8]/46,XX[12] |
| 3442 | Normal | 46,XY[20] |
| 3558 | Trisomy 12 | 47,XX,der(6)t(3;6)(q25;q21),+12[20] |
| 3622 | Trisomy 12 | 47,XY,+12[4]/46,XY[16] |
| 3628 | Del13q | 46,XX,del(13)(q12q14)[9]/46,XX[11] |
| 3634 | Trisomy 12 | 46,XX,+12,del(14)(q32)[14]/46,XX[6] |
| 3697 | Trisomy 12 | 47,XY,+12[13]/46,XY[7] |
| 3709 | Del13q | 46,XY,del(13)(q12q14),del(14)(q32)[7]/46,XY[13] |
| 3825 | Normal | 46,XY,del(14)(q22q32)[18]/46,XY[2] |
| 3917 | Del13q | 46,X,-Y,+3[5]/46,idem,del(13)(q12q14)[9]/46,XY[6] |
| 3929 | Del11q. del13q | 46,XY,del(11)(q22q23)[5]/46,idem,del(13)(q12q14)[8]/46,idem,del(14)(q32)[4]/46,XY[3] |
| 5002 | Del13q | 46,XY,del(13)(q14q22) [5]/46,XY[15] |
| 5004 | Del11q, del13q | 46,XY,del(11)(q13)[7]/46,XY,idem,del(13)(q14q22)[4]/46,XY[9] |
| 5006 | Trisomy 12 | 47,XX,+12[11]/46,XX[10] |
| 5008 | Normal | 45,XX,der(22)t(15;22)(q21;p11)[4]/45,idem,add(8)(p23)[10]/46,XX,del(6)(q22),-7,+10, add(10)(p15),-11,add(12)(q24),+17,+2mar[cp2]/46,XX[4] |
| 5010 | Del13q | 46,XY,del(13)(q14q22)[2]/46,XY[18] |
| 5020 | Del13q | 46,XY,del(13)(q14)[2]/45,idem,del(5)(q15),der(7)(p22),add(11)(q25),t(11;14)(q13;q32),-12,add(15)(q26),-22,+mar[4]/46,XY[16] |
| 5022 | Normal | 46,XY,del(14)(q32)[2]/46,XY[18] |
| 5024 | Del11q | 46,XX,add(10)(p15),del(11)(q21)[3]/46,XX[14] |
